# Incidentally detected breast lesions: a pictorial essay of malignant and benign findings

**DOI:** 10.31744/einstein_journal/2025RW0681

**Published:** 2025-05-30

**Authors:** Bruna Mayumi Takaki Tachibana, Rodrigo Marques Carneiro, Iviny Yonekura, Renato Leme de Moura Ribeiro, Ana Cláudia Silveira Racy, Érica Elisângela Francolin Federicci

**Affiliations:** 1 Hospital Israelita Albert Einstein São Paulo SP Brazil Hospital Israelita Albert Einstein, São Paulo, SP, Brazil.

**Keywords:** Breast neoplasms, Incidentalomas, Incidental findings, Diagnostic imaging, Tomography, X-ray computed, Positron emission tomography computed tomography

## Abstract

Incidentalomas are lesions incidentally identified in patients undergoing imaging for unrelated reasons. Breast incidentalomas have a high correlation with malignancy, occurring in over 40% of cases. Consequently, their interpretation poses significant challenges for radiologists who must discern the findings that may present a potential risk to the patient. This pictorial review presents different incidental breast lesions revealed through cross-sectional imaging. We further review key concepts related to such incidentalomas.

## INTRODUCTION

An incidentaloma is an unexpected finding detected in an imaging examination performed for an unrelated reason. The frequency of incidental findings is increasing due to the expanded use of cross-sectional imaging in routine practice, enhanced image quality, and improved access to modern imaging technologies.^(1)^ Consequently, radiologists frequently come across unexpected findings, even outside the anatomical regions of main interest; however, managing these findings can be challenging.

Unexpected breast lesions may be found in the cross-sectional chest and abdominal images and may include all or some of the mammary tissue. Management of incidental findings is important to avoid unnecessary imaging and surgery and to avoid increase in a patient's anxiety while accurately detecting potentially malignant lesions.^(2)^

A crucial aspect to emphasize incidentalomas is ensuring that patients follow up on relevant findings to reach a definitive diagnosis. It is essential to establish a structured process for monitoring and follow-up, typically involving communication with the referring physician or directly with the patient. In this article, we present various examples of incidental breast lesions identified through cross-sectional imaging, review key concepts associated with these findings, and describe the workflow employed for the management and follow-up of incidental findings in our Radiology Department.

## DISCUSSION

The estimated prevalence of breast incidentalomas in cross-sectional imaging ranges from 0.1-7.6% on CT, 0.01-9.3% on MRI, and approximately 0.4% on PET. Most of these prevalence data are derived from retrospective studies, analyzing explicitly documented information in radiologists’ reports, indicating that these figures are likely underestimated.^(2)^

**Figure 1:**
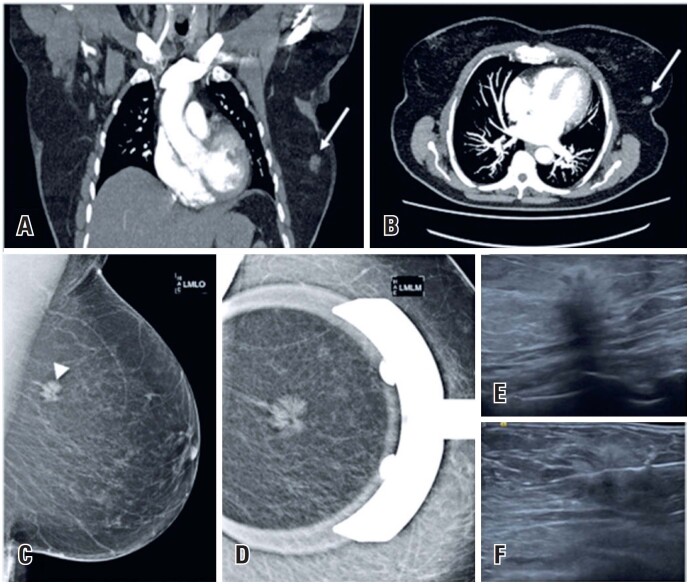
A 72-year-old woman arrived at to the emergency department with sudden shortness of breath and elevated serum D-dimer. Acute pulmonary embolism was suspected. (A and B) A chest computed tomography angiography (CTA) scan was requested and revealed no abnormalities, except for an incidental lesion on the left breast (arrows). (C and D) Mammography revealed an irregular spiculated mass - *BI-RADS 4* (arrowhead). (E and F) The lesion was identified on ultrasound and a US-guided core biopsy confirmed *invasive ductal carcinoma*

**Figure 2:**
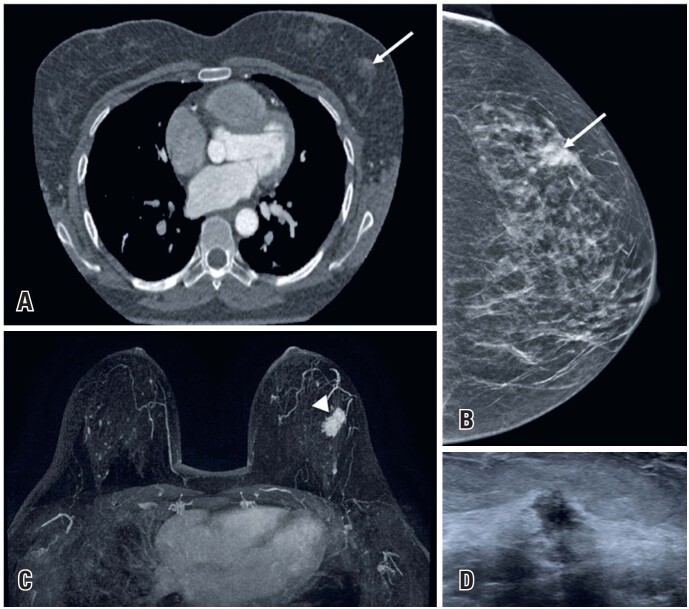
A 49-year-old female underwent a routine coronary CTA. (A) No coronary abnormalities were observed. However, an asymmetric density was identified in the left breast (arrow). (B) Mammography revealed an irregular spiculated mass, classified as BI-RADS 4 (arrow). (C) The lesion was evident on the MRI T1-weighted gadolinium-enhanced MIP sequence (arrowhead). (D) Ultrasound demonstrated a hypoechoic, irregular, angulated mass. Subsequent biopsy confirmed the diagnosis of invasive ductal carcinoma

**Figure 3:**
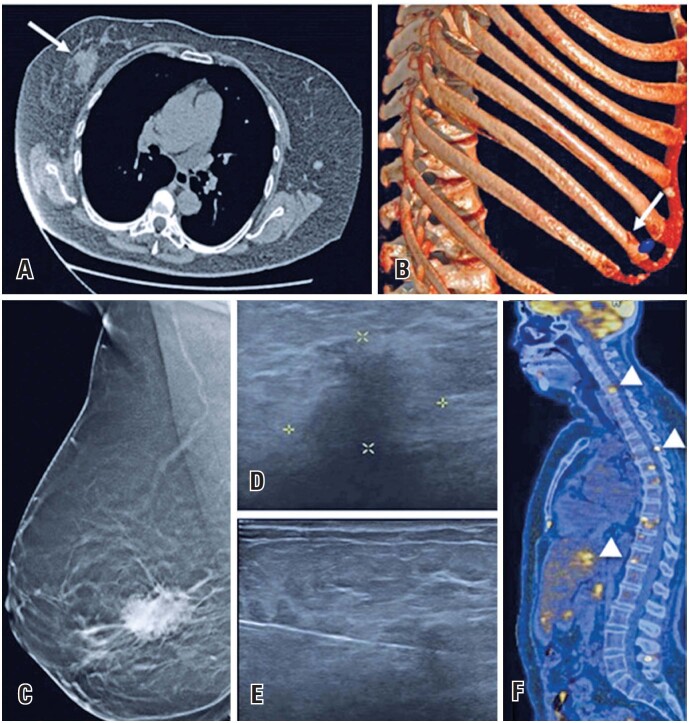
A 69-year-old woman presents to the emergency department with complaints of chest pain after a massage. (A and B) She underwent a chest computed tomography (CT), which revealed a rib fracture and an irregular density in the right breast (arrows). (C) Further investigation with mammography demonstrated an irregular spiculated mass —*BIRADS 4*. (D and E) Ultrasound-guided core biopsy revealed a *lobular carcinoma.* (F) A positron emission tomography (PET)-CT scan showed also hypermetabolic metastatic bone and liver lesions

Breast incidentalomas have a higher likelihood of malignancy compared to those found in other organs. An umbrella review encompassing 11 meta-analyses across 10 different organs found that 42% of all incidental breast findings were malignant neoplasms.^(1)^ Patients previously diagnosed with neoplasms were more likely to have malignant incidentalomas. The most associated factor was advanced age.^(1)^ Falomo et al.^(3)^ reported that, among patients with breast incidentalomas, the rate of malignancy was significantly higher in those with PET-detected lesions (55%) compared to those with CT- (35%) and MRI-detected (8%) lesions (Figures 1 to 3).

**Figure 4:**
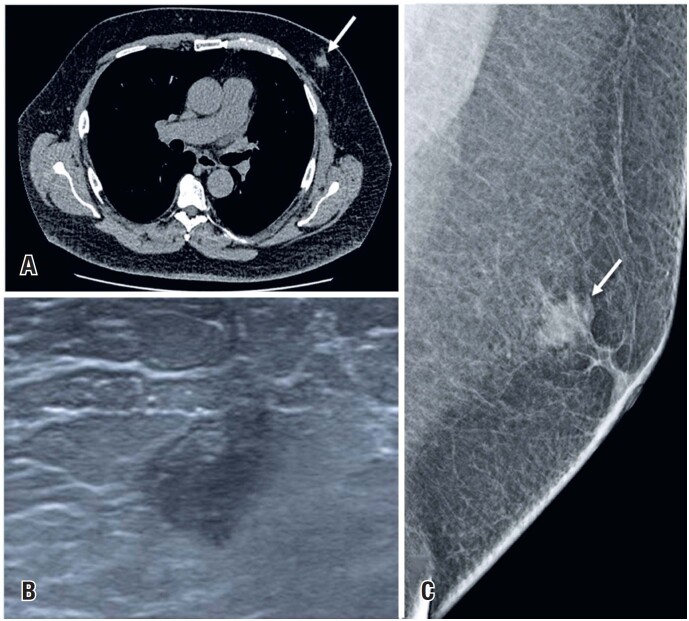
A 63-year-old man underwent a chest CT for a chronic cough. (A) The CT scan incidentally identified an irregular mass in the left breast (arrow). (B) On ultrasound showed the hypoechoic irregular mass. (C) Mammography showed an irregular indistinct mass—*BI-RADS 4*. The biopsy confirmed *invasive ductal carcinoma*

**Figure 5:**
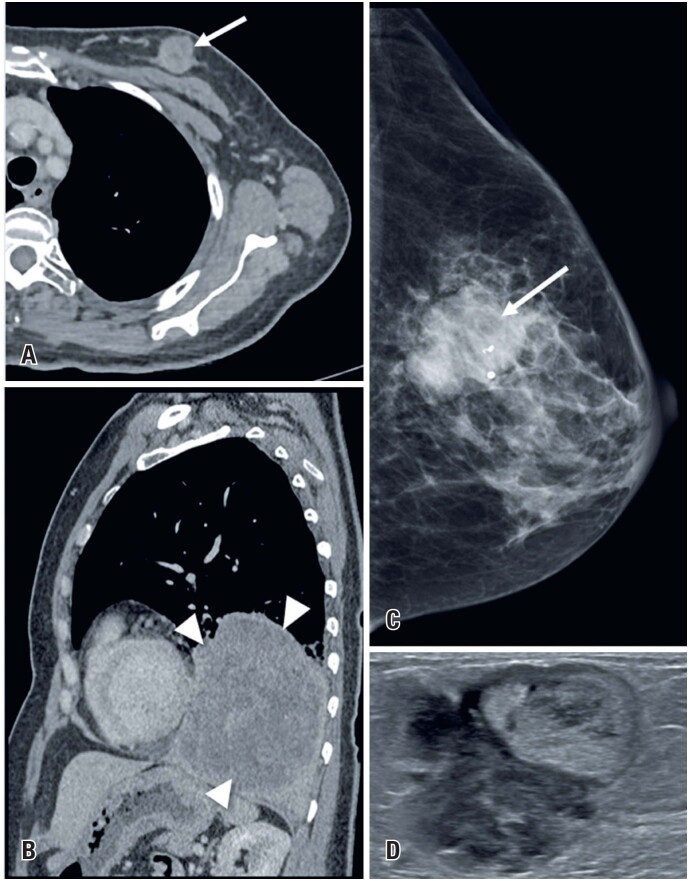
A 61-year-old woman undergoing treatment for small cell lung carcinoma had a follow-up chest CT. (A and B) In addition to the massive left lung lesion (arrowheads), a round mass was visualized in the left breast (arrow). (C) Mammography, showed an irregular obscured mass (arrow) classified as BI-RADS 4. (D) On ultrasound, it appeared as a solid-cystic lesion. A biopsy confirmed metastatic lung carcinoma

**Figure 6:**
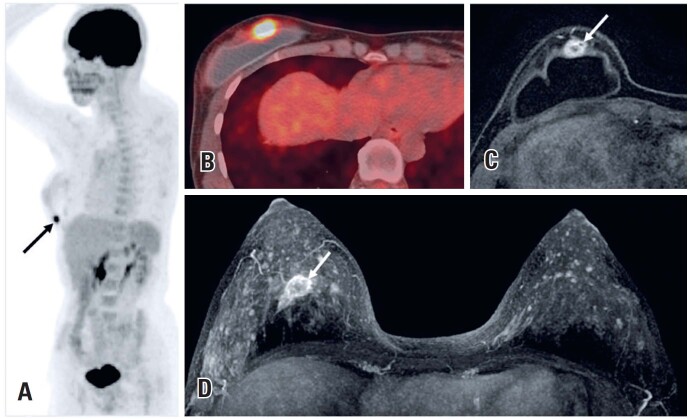
A 44-year-old woman under investigation for non-Hodgkin lymphoma underwent PET-CT (A and B). The PET-CT revealed increased uptake in the right breast (arrow). (C and D). Magnetic resonance imaging demonstrated an irregular mass with rim enhancement classified as BI-RADS 4. Breast biopsy confirmed invasive ductal carcinoma

**Figure 7:**
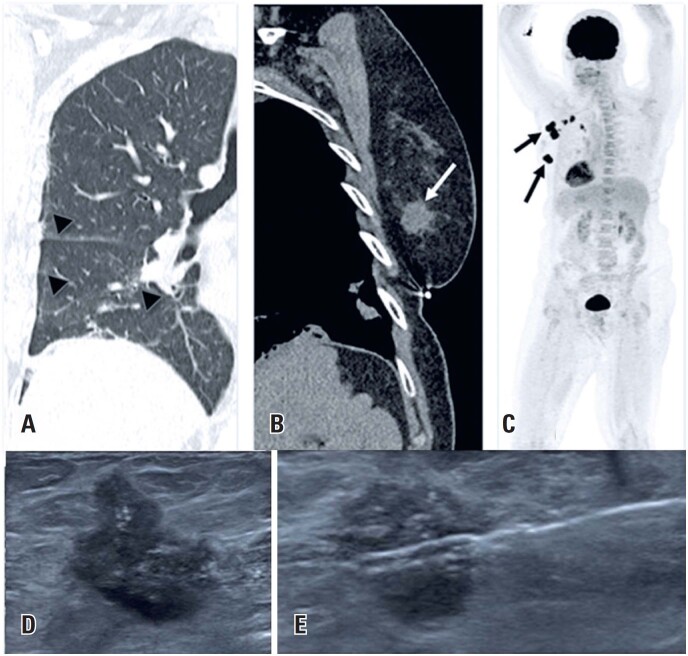
A 69-year-old woman presented to the emergency room with dyspnea and a positive COVID-19 polymerase chain reaction test. (A and B) A chest CT scan showed ground-glass opacities compatible with pulmonary viral infection (arrowheads) and an irregular left breast mass (arrow). (C) After recovery from COVID-19, a PET-CT revealed hypermetabolic lesions in the breast and axillary lymph nodes (arrows). (D and E) A core biopsy confirmed invasive ductal carcinoma

**Figure 8:**
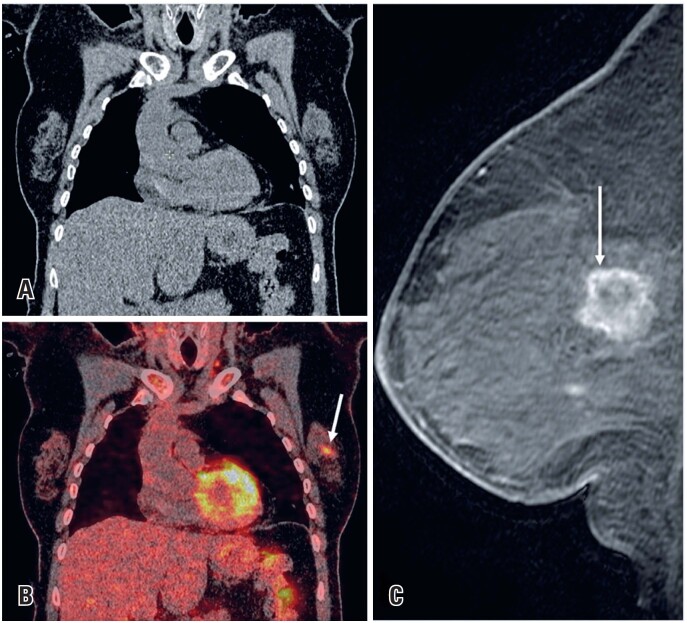
A 65-year-old woman with Lynch syndrome type II was being investigated for an ovarian neoplasm. (A and B) A PET-CT scan identified a hypermetabolic area in the left breast (arrow). A core biopsy confirmed invasive ductal carcinoma. (C) A subsequent breast MRI sagittal T1 - weighted contrast-enhanced imaging showed an irregular breast mass with rim enhancement and washout (arrow)

Due to their relatively high correlation with malignancy, breast incidentalomas must be meticulously documented by the radiologist.^(4-6)^ The report should include imaging characteristics, the patient's medical history (such as postoperative changes), and comparisons with previous examinations (if available); these aspects will help subsequent evaluations.^(7)^ Any breast mass incidentally found on cross-sectional imaging requires additional validation of benignity, like a demonstration of long-term stability or additional breast imaging.^(8)^ Most breast lesions will require further investigation with mammography and targeted ultrasound in women over the age of 40.^(2)^ (Figures 4 to 8).

**Figure 9:**
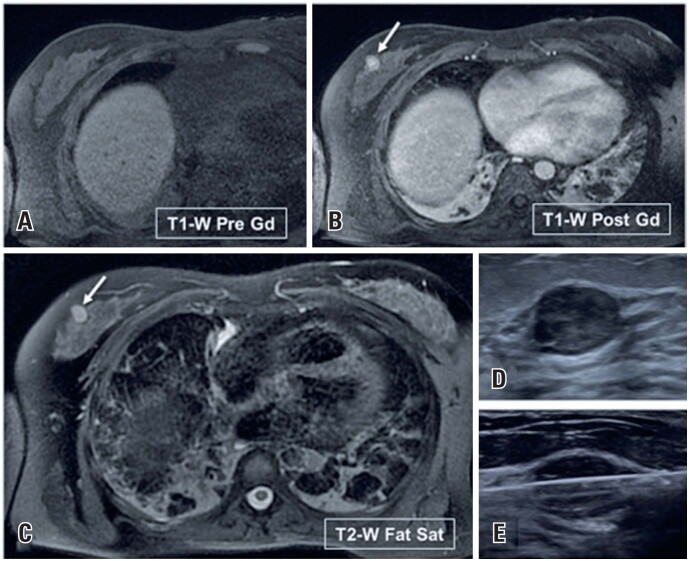
A 44-year-old woman with Crohn's disease underwent an abdominal MRI to evaluate a perianal fistula. (A to C) The MRI incidentally detected a vascularized mass in the right breast (arrows). (D and E) On ultrasound, the lesion appeared as an oval, circumscribed mass. A biopsy confirmed a Phyllodes tumor

**Figure 10:**
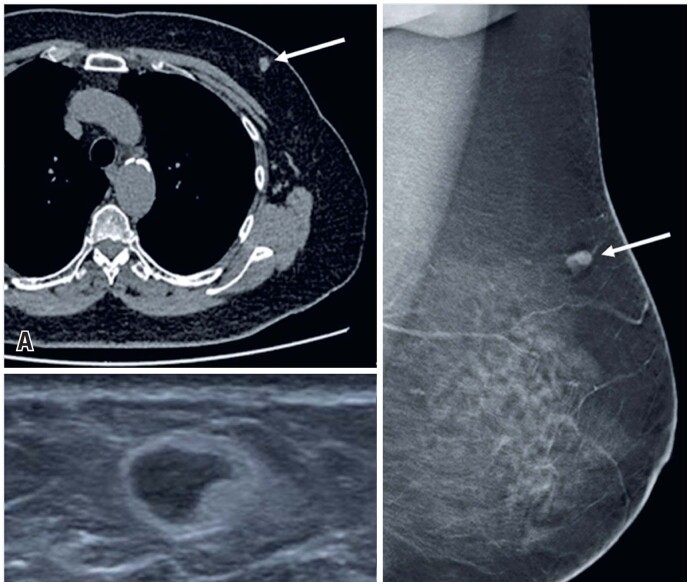
A 68-year-old woman underwent a chest CT for colon tumor staging. (A) The study identified a mass in the union of the upper quadrants of the left breast (arrow). (B) A breast ultrasound revealed an oval-shaped nodule. (C) Mammography showed a nodule suggestive of an atypical intramammary lymph node (arrow). The anatomopathological result was adipose tissue with fibroblastic reaction to hematologic material (*hematoma in organization*)

Although whole body CT and MRI are not highly sensitive for detecting breast cancer, most scans can reveal features that aid in stratifying the risk of malignancy of the lesions. Spiculated margins, irregular shape, mass-like enhancement, FDG-avidity (on PET-CT), skin thickening or retraction, trabecular thickening, and coexisting axillary lymphadenopathy often predict malignancy.^(8)^ In contrast, microcalcifications that are highly associated with malignancy on mammography, are below the CT spatial resolution. Most calcifications seen on CT are benign and include fat necrosis, oil cysts, degenerating fibroadenomas, and suture or vascular calcifications. The presentation of calcifications frequently associated to benignity include lucent-centered, round, eggshell or rim, coarse or popcorn-like, and large rodlike^(7)^ (Figures 9 and 10).

Al-katib et al.^(8)^ proposed an algorithm for management of incidentally detected breast findings in CT scans of patients without previous mammography. Macrocalcifications and macroscopic fat were defined as *benign*; skin thickening and round masses were defined as *indeterminate*, prompting additional validation of stability (at least 2 years) or dedicated breast imaging; and masses with irregular shapes or spiculated margins, mass-like enhancement, and unilateral axillary lymphadenopathy were defined as *highly suspicious*, requiring dedicated breast imaging.

To the best of our knowledge, there are currently no specific guidelines available for the management of breast incidentalomas. In our institution, the Radiology Department has established a protocol for following up on all patients with incidental findings. This process aims to ensure, whenever possible, that a definitive diagnosis is reached or that the lesions identified in the exams are appropriately monitored. Our protocol involves the radiologist, who records the incidental findings in the Radiology Information System; the physician responsible for communicating the finding to the patient, who may be the referring physician or the radiologist; and the quality and processes team, which monitors the progress of all cases until a final outcome is achieved.

Currently, more than 3 years after the beginning of the COVID-19 pandemic, breast cancer screenings were postponed or canceled for many patients due to the pandemic.^(9,10)^ The unfortunate consequence of such decisions resulted in delayed diagnosis, potentially leading to patients presenting with more advanced disease and worse clinical outcomes, including both expected and unexpected breast findings. Hence, it even more important to follow the correct diagnosis, interpretation and management of the incidentalomas found in other exams.

## CONCLUSION

Incidental breast findings can present in various forms, depending on the cross-sectional imaging method used. In the absence of confirmed long-term stability or prior characterization in other exams, most breast incidentalomas necessitate dedicated breast imaging and biopsy for specific suspicious lesions. Radiologists must effectively manage these incidental findings to prevent misinterpretation, which could lead to unnecessary investigations (causing patient anxiety and increasing economic burden) or, conversely, result in a missed critical diagnosis.
